# Green tea polyphenol extract attenuates lung injury in experimental model of carrageenan-induced pleurisy in mice

**DOI:** 10.1186/1465-9921-6-66

**Published:** 2005-06-29

**Authors:** Rosanna Di Paola, Emanuela Mazzon, Carmelo Muià, Tiziana Genovese, Marta Menegazzi, Raffaela Zaffini, Hisanory Suzuki, Salvatore Cuzzocrea

**Affiliations:** 1 Department of Clinical and Experimental Medicine and Pharmacology, Torre Biologica, Policlinico Universitario, Messina, Italy; 2 Biochemistry Division, Department of Neuroscience and Vision, University of Verona, Verona, Italy

**Keywords:** green tea extract, carrageenan-induced pleurisy, neutrophils infiltration, lung injury

## Abstract

Here we investigate the effects of the green tea extract in an animal model of acute inflammation, carrageenan-induced pleurisy. We report here that green tea extract (given at 25 mg/kg i.p. bolus 1 h prior to carrageenan), exerts potent anti-inflammatory effects in an animal model of acute inflammation in vivo.

Injection of carrageenan (2%) into the pleural cavity of mice elicited an acute inflammatory response characterized by fluid accumulation in the pleural cavity that contained many neutrophils (PMNs), an infiltration of PMNs in lung tissues and increased production of nitrite/nitrate, tumour necrosis factor alpha. All parameters of inflammation were attenuated by green tea extract treatment. Furthermore, carrageenan induced an up-regulation of the adhesion molecule ICAM-1, as well as nitrotyrosine and poly (ADP-ribose) synthetase (PARS) formation, as determined by immunohistochemical analysis of lung tissues. Staining for the ICAM-1, nitrotyrosine, and PARS was reduced by green tea extract.

Our results clearly demonstrate that treatment with green tea extract exerts a protective effect and offers a novel therapeutic approach for the management of lung injury.

## Introduction

The role of oxyradical formation in various forms of inflammation is well established [1] Reactive oxygen species (ROS) are associated with the inflammatory response and frequently they contribute to the tissue damaging effects of inflammatory reactions [2-4]. ROS formation and degradation are key components of the metabolism of aerobic organisms. Certain levels of ROS are required for normal cell functions, but if in surplus, they will cause oxidative stress [5-7]. ROS like superoxide, hydrogen peroxide and lipid hydroperoxides can regulate the activities of several kinases, transcription factors, cell death machinery and proteins such as COX-2 and iNOS [8,9].

Recent data demonstrate that the expression of the inducible isoform of nitric oxide (NO) synthase also plays important pathogenetic roles in various models of inflammation [10-12]. Peroxynitrite, a cytotoxic oxidant species formed from the reaction of NO and superoxide [13], may mediate part of the oxidative injury associated with simultaneous production of NO and oxyradicals. Peroxynitrite formation has been demonstrated in various inflammatory disorders [14,15] and in circulatory shock [16]. Peroxynitrite is a potent oxidant, and therefore it is conceivable that endogenous antioxidant mechanisms counteract its toxicity. In *in vitro *studies, it has been established that antioxidants such as glutathione, ascorbic acid, and alpha-tocopherol are scavengers of peroxynitrite and inhibitors of its oxidant capacity [17,18].

Green tea – a minimally processed product of the same plant that gives us black and oolong teas – is rich in powerful antioxidant compounds called polyphenols. The polyphenols found in tea are more commonly known as flavanols or catechins and comprise 30–40 percent of the extractable solids of dried green tea leaves. The main catechins in green tea are epicatechin, epicatechin-3-gallate, epigallocatechin, and epigallocatechin-3- gallate (EGCG), with the latter being the highest in concentration. Green tea polyphenols have demonstrated significant antioxidant, anticarcinogenic, anti-inflammatory, thermogenic, probiotic, and antimicrobial properties in numerous human, animal, and *in vitro *studies [19,20]. Recently it has been showed that green tea polyphenols inhibited tumour necrosis factor-alpha induction in macrophages by attenuating nuclear factor-kβ NF-Kβ) activation [21]. Similarly [22] showed that EGCG inhibits lipopolysaccaride (LPS) – stimulated nitric oxide production and inducibile nitric oxide synthase gene expression in peritoneal macrophages by decreasing NF-κβ activation. These studies provide significant evidence that green tea polyphenols have anti-inflammatory effects. Lung inflammation is characterised by T-cell rich infiltrates and enhanced expression of pro-inflammatory cytokines. The signalling pathway of IFN-γ, secreted by type-1 helper lymphocyte (Th-1), lead to the activation of signal transducer and activator of transcription-1 (STAT-1) [23]. Moreover, IFN-γ is involved in the induction of iNOS and ICAM-1 gene expression by the activation of STAT-1 transcription factor [24,25]. Thus, upregulation of STAT-1 activity could play a key role in the pathogenesis of carrageenan-induced pleurisy. STAT-1 are activated by phosphorylation on conserved tyrosine and serine residues by the Janus kinases (JAKs) and MAP kinase families respectively, which allow the STAT-1 to dimerise and translocate to the nucleus and there by regulate gene expression [23]. Previously, we demonstrated, in some epithelial cell cultures, the inhibitory effect of EGCG on iNOS induction by preventing STAT-1 phosphorylation and activation [26].

In this study we investigated the role of Green tea extract in rodent model carrageenan-induced pleurisy.

This experimental model has been widely used to investigate the pathophysiology of acute inflammation and also to evaluate the efficacy of drugs in inflammation. Injection of carrageenan into the pleural space leads to pleurisy, infiltration by polymorphonuclear leukocytes (PMN), and lung injury. In this study, we have investigated the effect of the green tea on: PMN infiltration [myeloperoxidase (MPO) activity]; STAT-1 activity (by EMSA), up-regulation of ICAM-1 (by immunohistochemistry); the nitration of tyrosine residues (an indicator of the formation of peroxynitrite) (by immunohistochemistry) and lung damage (histology).

## Materials and methods

### Animals

Mice (4–5 weeks old, 20–22 g) were purchased from Jackson Laboratories (Harlan Nossan, Italy). The animals were housed in a controlled environment and provided with standard rodent chow and water. Animal care was in compliance with Italian regulations on protection of animals used for experimental and other scientific purposes (D.M. 116192) as well as with the EEC regulations (O.J. of E.C. L 358/1 12/18/1986).

### Green tea extract

Green tea extract (GTE) was a kind gift of Indena (Milano, Italy), and it was defined by the producer as having a polyphenolic content of 75 ± 5% with the major constituent being epigallocatechin-3-gallate at 62% and the minor ones being epicatechin-3-gallate, epigallocatechin and epicathechin.

### Carrageenan-induced pleurisy

Mice were anaesthetised with isoflurane and submitted to a skin incision at the level of the left sixth intercostals space. The underlying muscle was dissected and saline (0.1 ml) or saline containing 2%λ-carrageenan (0.1 ml) was injected into the pleural cavity. The skin incision was closed with a suture and the animals were allowed to recover. At 4 h after the injection of carrageenan, the animals were killed by inhalation of CO_2_. The chest was carefully opened and the pleural cavity rinsed with 1 ml of saline solution containing heparin (5 U/ml) and indomethacin (10 μg/ml). The exudate and washing solution were removed by aspiration and the total volume measured. Any exudate, which was contaminated with blood, was discarded. The amount of exudate was calculated by subtracting the volume injected (1 ml) from the total volume recovered. The leukocytes in the exudate were suspended in phosphate-buffer saline (PBS) and counted with an optical microscope in a Burker's chamber after Blue Toluidine staining.

### Experimental groups

Mice were randomly allocated into the following groups: (i) *CAR + saline group*. Mice were subjected to carrageenan-induced pleurisy (*N *= 10), (ii) *Green Tea group *Same as the *CAR + saline group *but Green Tea (25 mg/kg i.p) were administered 1 h prior to carrageenan (*N *= 10), (iii) *Sham+saline group*. Sham-operated group in which identical surgical procedures to the CAR group was performed, except that the saline was administered instead of carrageenan (*N *= 10), (iv) *Sham + Green Tea group*. Same as the Sham+saline group but Green Tea (25 mg/kg i.p) were administered 1 h prior to carrageenan (N = 10). The doses of Green Tea used here to reduce acute lung injury have been reported by us to reduce the tissue injury caused by ischemia-reperfusion in the gut (dose-response curve study) (Muià *et al *submitted 2005).

### Determination of myeloperoxidase activity

Myeloperoxidase (MPO) activity, an indicator of polymorphonuclear leukocyte (PMN) accumulation, was determined as previously described [27]. At 4 h after intrapleural injection of carrageenan lung tissues, were obtained and weighed. Each piece of tissue was homogenised in a solution containing 0.5% hexa-decyl-trimethyl-ammonium bromide dissolved in 10 mM potassium phosphate buffer (pH 7) and centrifuged for 30 min at 20,000 × g at 4°C. An aliquot of the supernatant was then allowed to react with a solution of tetra-methyl-benzidine (1.6 mM) and 0.1 mM H_2_O_2_. The rate of change in absorbance was measured spectrophotometrically at 650 nm. MPO activity was defined as the quantity of enzyme degrading 1 μmol of peroxide min at 37°C and was expressed in mill units per gram weight of wet tissue.

### Measurement of TNF-α levels

TNF-α levels were evaluated in the exudates at 4 h after the induction of pleurisy by carrageenan injection. The assay was carried out by using a colorimetric, commercial ELISA kit (Calbiochem-Novabiochem Corporation, USA).

### Measurement of nitrite/nitrate

Nitrite/nitrate (NOx) production, an indicator of NO synthesis, was measured in pleural exudate. At the first nitrate in the supernatant was incubated with nitrate reductase (0.1 U/ml) and NADPH (1 mM) and FAD (50 μM) at 37°C for 15 min. Then followed another incubation with LDH (100 U/ml) and sodium pyruvate (10 mM) for 5 min. The nitrite concentration in the samples was measured by the Griess reaction, by adding 100 μl of Griess reagent (0.1% naphthylethylenediamide dihydrochloride in H_2_O and 1% sulphanilamide in 5% concentrated H_2_PO_4_; vol. 1: 1) to 100 μl samples. The optical density at 550 nm (OD_550_) was measured using ELISA microplate reader (SLT- Lab instruments Salzburg, Austria). Nitrate concentrations were calculated by comparison with OD_550 _of standard solutions of sodium nitrate prepared in saline solution.

### Immunohistochemical localisation of ICAM-1, PAR and Nitrotyrosine

At 4 h after carrageenan administration, the lungs were fixed in 10% buffered formaldehyde and 8 μm sections were prepared from paraffin embedded tissues. After deparaffinization, endogenous peroxidase was quenched with 0.3% H_2_O_2 _in 60% methanol for 30 min. The sections were permeabilized with 0.1% Triton X-100 in PBS for 20 min. Non-specific adsorption was minimised by incubating the section in 2% normal goat serum in phosphate buffered saline for 20 min. Endogenous biotin or avidin binding sites were blocked by sequential incubation for 15 min with avidin and biotin. The sections were then incubated overnight with primary anti-ICAM-1 antibody (1:500), with 1:1000 dilution of primary antinitrotyrosine antibody (DBA), and anti-PAR antibody (1:500) or with control solutions. Controls included buffer alone or non-specific purified rabbit IgG.

To confirm that the immunoreaction for the nitrotyrosine was specific, some sections were also incubated with the primary antibody (anti-nitrotyrosine) in the presence of excess nitrotyrosine (10 mM) to verify the binding specificity. To verify the binding specificity for PARS, sections were also incubated with only the primary antibody (no secondary) or with only the secondary antibody (no primary). In these situations, no positive staining was found in the sections, indicating that the immunoreaction was positive in all the experiments carried out.

Immunocytochemistry photographs (n = 5) were assessed by densitometry. The assay was carried out by using Optilab Graftek software on a Macintosh personal computer (CPU G3-266).

### Histological examination

Lung biopsies were taken at 4 h after injection of carrageenan. The biopsies were fixed for 1 wk in buffered formaldehyde solution (10% in PBS) at room temperature, dehydrated by graded ethanol and embedded in Paraplast (Sherwood Medical, Mahwah, N.J.). Tissue sections (thickness 7 μm) were deparaffinized with xylene, stained with trichromic Van Gieson, and studied using light microscopy (Dialux 22 Leitz). Blood was passed on the slide, fixed at 37°C, stained with May Grunward-Giensa, and studied using light microscopy.

### Electrophoretic mobility shift assay

The lung samples have been collected in liquid nitrogen and stored at -80°C until use. Nuclear extracts have been prepared according to [28] in the presence of 10 μg/ml leupeptin, 5 μg/ml antipain and pepstain, and 1 mM PMSF (Sigma-Aldrich Company, Milan, Italy). Protein concentration in the nuclear extracts was determined using the method of [29]. Ten μg of nuclear extract have been incubated at room temperature for 20 min with (2–5 × 10^4^cpm) of ^32^P-labeled double stranded oligonucleotide, containing the STAT-1 binding site (sis-inducible factor-binding recognition element, SIE/M67) from the c-Fos promoter (5'-GTCGACATTTCCCGTAAATCG-3'), the PARP-1 binding site (5'-TTCCTTGCCCCTCCCATTTTTC-3') from the Reg promoter [30] or the SP-1 consensus sequence (5'GGGGCGGGGC-3', Santa Cruz Biotechnology, CA) in a 15 μl of binding reaction buffer (20 mM HEPES, pH 7.9, 50 mM KCl, 10% glycerol, 0.5 mM DTT, 0.1 mM EDTA, 2 μg poly(dI-dC), 1 μg salmon sperm DNA). Products have been fractioned on a non denaturing 5% polyacrilamide gel in TBE (Tris-Borate-EDTA buffer, 0.5X). The intensity of the retarded bands has been measured with a Phosphorimager (Molecular Dynamic, Milan, Italy).

### Materials

Unless otherwise stated, all compounds were obtained from Sigma-Aldrich Company (Milan, Italy). Primary monoclonal ICAM-1 (CD54) for immunoistochemistry was purchases by Pharmingen. Reagents and secondary and nonspecific IgG antibody for immunohistochemical analysis were from Vector Laboratories InC. Primary monoclonal anti-poly (ADP-ribose) antibody was purchased by Alexis. All other chemicals were of the highest commercial grade available. All stock solutions were prepared in non pyrogenic saline (0.9% NaCl; Baxter Healthcare Ltd., Thetford, Norfolk, U.K.).

### Data analysis

All values in the figures and text are expressed as mean ± standard error (s.e.m.) of the mean of *n *observations. For the *in vivo *studies *n *represents the number of animals studied. In the experiments involving histology or immunohistochemistry, the figures shown are representative of at least three experiments performed on different experimental days. The results were analysed by one-way ANOVA followed by a Bonferroni *post-hoc *test for multiple comparisons. A *p*-value less than 0.05 was considered significant.

## Results

### Effects of green tea extract in carrageenan-induced pleurisy

**Figure 1:**
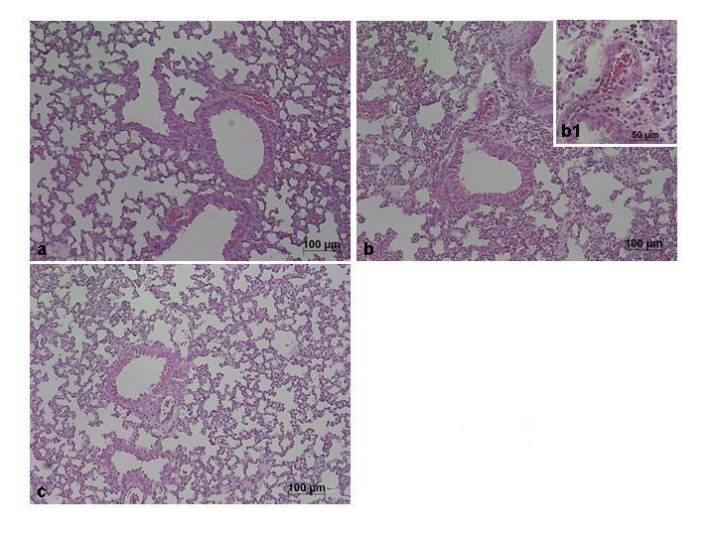
**Effect of GTE on lung injury**. When compared with lung sections taken from control animals (A), lung sections from carrageenan-treated mice (B) demonstrate interstitial haemorrhage and polymorphonuclear leukocyte accumulation (B1). Lung sections from a carrageenan-treated mice that received GTE (C) exhibit reduced interstitial haemorrhage and a lesser cellular infiltration. Figure is representative of all the animals in each group.

**Figure 2:**
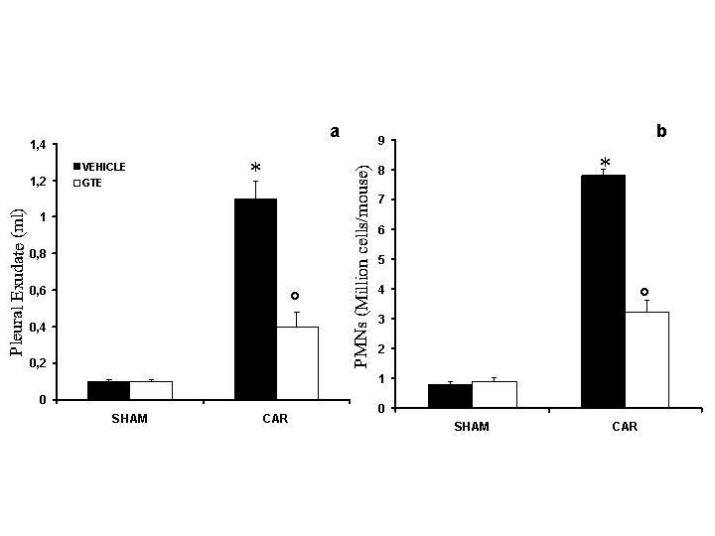
**Effect of GTE on carrageenan-induced inflammation**. The increase in volume exudate (A) and accumulation of polymorphonuclear cells (PMNs, B) in pleural cavity 4 h after carrageenan injection was inhibited by GTE. Data are means ± SEM of 10 mice for each group. *P < 0.01 vs. sham. °*P *< 0.01 vs. carrageenan.

Histological examination of lung sections revealed significant tissue damage (Fig. 1B). Thus, when compared with lung sections taken from saline-treated animals (Fig. 1A), histological examination of lung sections of mice treated with carrageenan showed oedema, tissue injury (Fig 1B), and infiltration of the tissue with neutrophils (PMNs) (Fig. 1B1). GTE significantly reduced the degree of injury as well as the infiltration of PMNs (Fig. 1C). Furthermore, injection of carrageenan into the pleural cavity of mice elicited an acute inflammatory response characterized by the accumulation of fluid (oedema) that contained large amounts of PMNs (Fig. 2A,B). Oedema and PMNs infiltration in pleural cavity were attenuated by the i.p. injection of GTE (Figs. 2A,B).

### Effect of green tea extract on TNF-α levels

**Figure 3:**
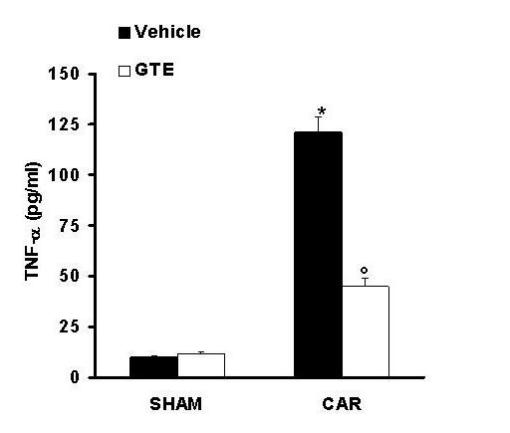
**Effect of GTE on TNF-α, level**. Pleural injection of carrageenan caused by 4 h an increase in the release of the pro-inflammatory cytokines, tumour necrosis factor alpha (TNF-α). GTE significantly inhibited TNF-α. Data are means ± SEM of 10 mice for each group. *P < 0.01 vs. sham. °*P *< 0.01 vs. carrageenan.

The levels of TNF-α were significantly elevated in the exudates from vehicle-treated mice at 4 h after carrageenan administration (Fig. 3). In contrast, the levels of this pro-inflammatory cytokine was significantly lower in carrageenan-treated mice treated with GTE (Fig. 3). No significant increased of TNF-α levels was observed in the exudates of sham-operated mice.

### Effects of green tea extract on MPO activity

**Figure 4:**
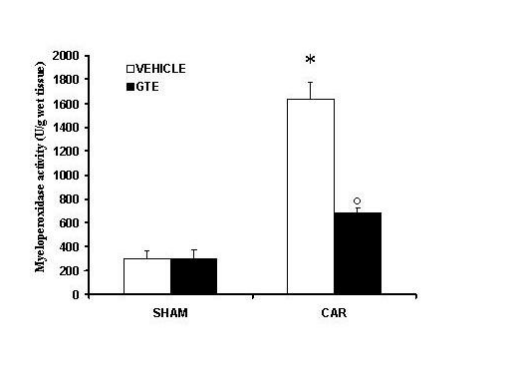
**Effect of GTE on myeloperoxidase (MPO) activity in the lung**. Within 4 h, pleural injection of carrageenan led to an increase in neutrophil accumulation in the lung (as measured by MPO activity) GTE treatment significantly inhibited neutrophil infiltration. Data are means ± SEM of 10 mice for each group. *P < 0.01 vs. sham. °*P *< 0.01 vs. carrageenan.

The above histological pattern of lung injury appeared to be correlated with the influx of leukocytes into the lung tissue. Therefore, we investigate the role of GTE on the neutrophils infiltration by measurement of the activity of myeloperoxidase. Myeloperoxidase activity was significantly elevated (p < 0.001) at 4 h after carrageenan administration in vehicle-treated mice (Fig. 4). In Mice treated with green tea extract lung myeloperoxidase activity was significantly reduced (p < 0.01) in comparison to those of vehicle-treated mice (Fig. 4).

### Effects of green tea extract on the expression of adhesion molecule (ICAM-1)

**Figure 5:**
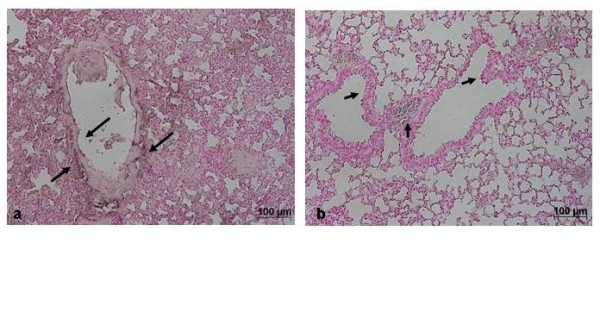
**Immunohistochemical localization of ICAM-1 in the lung**. Section obtained from carrageenan-treated mice showed intense positive staining for ICAM-1 (A, see arrows). The degree of bronchial epithelium (see arrows) staining for ICAM-1 (B) was markedly reduced in tissue section obtained from GTE-treated mice. Figure is representative of all the animals in each group.

**Figure 6:**
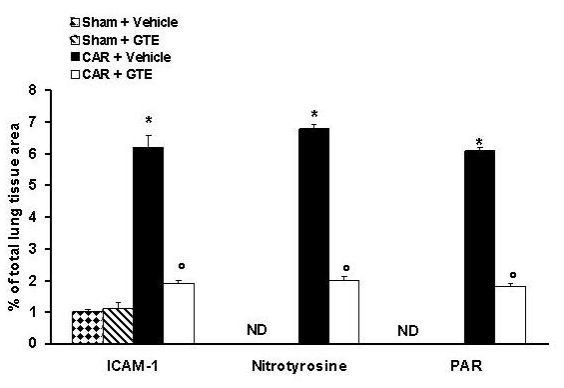
**Typical Densitometry evaluation**. Densitometry analysis of immunocytochemistry photographs (n = 5) for ICAM-1, Nitrotyrosine and PAR from lung was assessed. The assay was carried out by using Optilab Graftek software on a Macintosh personal computer (CPU G3-266). Data are expressed as % of total tissue area. ND: not detectable. *P < 0.01 vs. sham. °*P *< 0.01 vs. carrageenan.

Staining of lung tissue sections obtained from saline-treated mice with anti-ICAM-1 antibody showed specific staining along bronchial epithelium, demonstrating that ICAM-1 is constitutively expressed (data not shown). At 4 h after carrageenan injection, the staining intensity substantially increased along the bronchial epithelium (see arrows, Fig. 5A, 6). Sections from GTE-treated mice did not reveal any up-regulation of constitutively expressed ICAM-1, which was normally expressed in the epithelium (see arrows, Fig. 5B, 6). To verify the binding specificity for ICAM-1, some sections were also incubated with only the primary antibody (no secondary). In these situations, no positive staining was found in the sections, indicating that the immunoreaction was positive in all the experiments carried out.

### Effects of green tea extract on nitric oxide production

**Figure 7:**
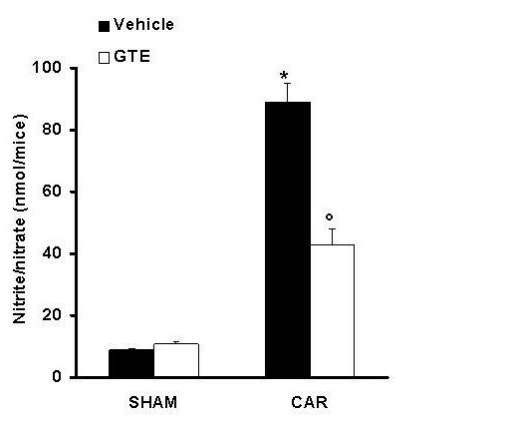
**Effect of GTE on NO production**. Nitrite and nitrate concentrations in pleural exudate at 4 h after carrageenan administration. Nitrite and nitrate levels in carrageenan-treated mice was significantly increased *vs*. sham group. GTE treatment significantly reduced the carrageenan-induced elevation of nitrite and nitrate levels. Data are means ± SEM of 10 mice for each group. *P < 0.01 vs. sham. °*P *< 0.01 vs. carrageenan.

The levels of NO_x _were significantly (*P *< 0.01) increased in the exudate from carrageenan-treated mice (Fig. 7). In contrast, levels of NO_x _were significantly lower in the exudate of mice treated with GTE (Fig. 7).

### Effects of green tea extract on nitrotyrosine and PARS

**Figure 8:**
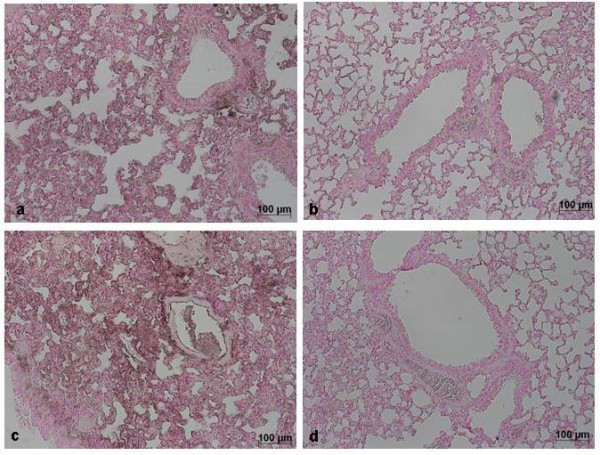
**Immunohistochemical localization for nitrotyrosine and PARS in the lung**. Immunohistochemistry for nitrotyrosine (A) and PARS (C) show positive staining along the vessels and in the bronchial epithelium from a carrageenan-treated mice. The intensity of the positive staining for nitrotyrosine (B) and PARS (C) was significantly reduced in the lung from GTE-treated mice. Figure is representative of all the animals in each group.

**Figure 9:**
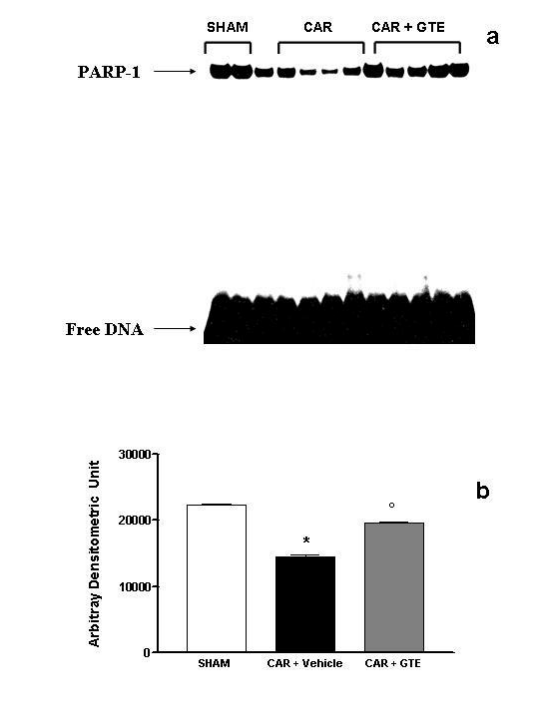
**Effect of GTE on PARP-1 activation**. (A) DNA binding activity of PARP-1 in sham operated, carrageenan-treated (CAR) and GTE pre-treated mice (CAR + GTE). Nuclear extracts (10 μg) from lung sample were incubated with a ^32^P-labeled double-stranded oligonucleotide containing binding sequence for PARP-1 and separated by nondenaturing PAGE. The specificity of the retarded bands was demonstrated by competition with 100-fold excess of specific unlabeled oligonucleotide (not shown). (B) The intensity of retarded bands (measured by phosphoimager) in carrageenan-treated mice was significantly increased *vs*. sham group. GTE treatment significantly reduced the carrageenan-induced elevation of PARP-1 activity. Data are means ± SEM of 5 mice for each group. *P < 0.01 vs. sham. °*P *< 0.001 vs. carrageenan.

At 4 h after carrageenan injection, lung sections were taken in order to determine the immunohistological staining for nitrotyrosine or PARS. Sections of lung from saline-treated mice did not reveal any immunoreactivity for nitrotyrosine or PARS within the normal architecture (data not shown). A positive staining for nitrotyrosine (Fig. 6, 8A) and PARS (Fig. 6, 8C) was localized primarily in the vessels and in the bronchial epithelium. GTE reduced the staining for both nitrotyrosine (Fig. 6, 8B) and PARS (Fig. 6, 8D). Therefore, no differences between groups were shown for SP-1 DNA binding activity (data not shown). It was also shown the DNA binding capacity of PARP-1 to the promoter sequence of the Reg gene [30]. The retarded bands of the carraggeenan-treated mice were reduced in comparison to those of vehicle-treated or GTE pre-treated mice (Fig. 9A, B)

### Effects of green tea extract on the STAT-1 activation

**Figure 10:**
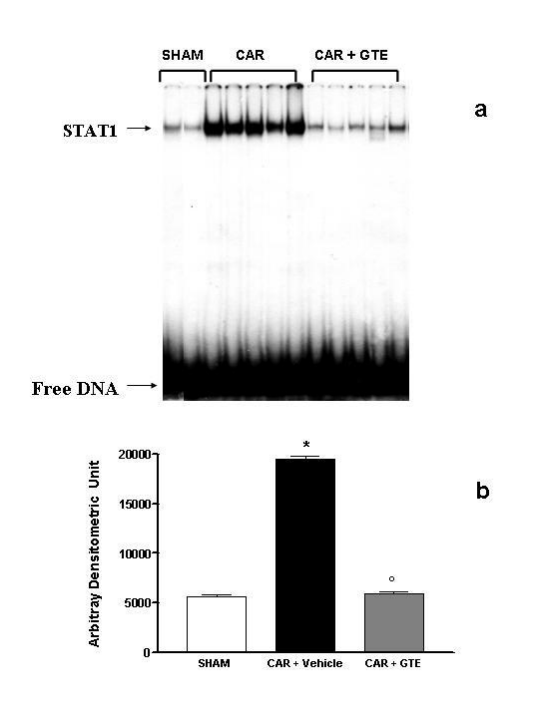
**Effect of GTE on STAT-1 activation**. (A) DNA binding activity of STAT-1 in sham operated, carrageenan-treated (CAR) and GTE pre-treated mice (CAR + GTE). Nuclear extracts (10 μg) from lung sample were incubated with a ^32^P-labeled double-stranded oligonucleotide containing binding sequence for STAT-1 and separated by nondenaturing PAGE. The specificity of the retarded bands was demonstrated by competition with 100-fold excess of specific unlabeled oligonucleotide (not shown). (B) The intensity of retarded bands (measured by phosphoimager) in carrageenan-treated mice was significantly increased *vs*. sham group. GTE treatment significantly reduced the carrageenan-induced elevation of STAT-1 activity. *P < 0.01 vs. sham. °*P *< 0.001 vs. carrageenan.

To examine the molecular mechanisms responsible for mediating the anti-inflammatory effects of GTE we measured, by EMSA, the changes in activation of the transcription factors STAT-1 and SP-1. DNA-binding activity of STAT-1 was significantly elevated at 4 h after carrageenan administration in vehicle-treated mice (Fig. 10A, B). In Mice treated with green tea extract lung STAT-1 activity was similar to those of sham-operated group and significantly reduced in comparison to those of vehicle-treated mice (Fig. 10A, B).

## Discussion

Polyphenols are the most significant group of tea components, especially the catechin group of the flavonols. The major tea catechins are EGCG, EGC, ECG, EC, (+)-gallocatechin, and (+)-catechin.

Many biological functions of tea polyphenols have been studied [31], including anti-inflammatory, antioxidative [32-34], antimutagenic [35], and anticarcinogenic [36] effects.

This study provides the evidence that pretreatment of mice with green tea extract attenuates 1) the development of carrageenan-induced pleurisy, 2) the infiltration of the lung with PMNs (histology and MPO activity), 3) the degree of lung injury (histology) caused by injection of carrageenan. All of these findings support the view that green tea extract attenuates the degree of acute inflammation in mice. What, then, is the mechanism by which green tea extract reduces acute inflammation?

The generation of oxidative and nitrosative species, which exert their effects both directly and indirectly, is a important contributor to inflammatory injury. The terms *oxidative *and *nitrosative *refer to the formation of reactive oxygen species (ROS), such as superoxide (O_2_^.-^), hydrogen peroxide (H_2_O_2_), and hydroxyl radicals, and reactive nitrogen species (RNS), such as nitric oxide (NO), peroxynitrite (ONOO^-^), and nitrogen dioxide.

Oxidants are generated as a result of the inflammatory response by phagocytic cells, such as mononuclear cells. Oxidants that are generated in excess of antioxidant defences or that are lacking in antioxidant defences can result in severe pulmonary inflammation leading to acute lung injury Additionally, NO plays a multifaceted role in mediating inflammatory processes [37]. Potential sources of NO in the lungs include expression of iNOS, activated neutrophils, [38] alveolar type-IIcells [39] endothelial cells [40] and airway cells [41]. It has been demonstrated that levels of NO_2_^-^, increase markedly during acute and chronic inflammation [42]. Recent study had demonstrated that green tea polyphenols inhibit NO production in peritoneal exudate (macrophage) cells [43] and EGCG inhibits lipopolysaccharide (LPS)-induced NO production and iNOS gene expression in isolated peritoneal macrophages by decreasing NF-KB activation [22]. In agreement with these observations in this study we shown that the treatment with green tea extract in vivo reduce NO formation.

Simultaneous generation of NO^. ^and O_2_^.- ^favours the production of a toxic reaction product, peroxynitrite anion (ONOO^-^)[13] and this product may account for some of the deleterious effects associated with NO^. ^production.

The pro-inflammatory and cytotoxic effects of ONOO- are numerous [44]. Peroxynitrite nitrosates tyrosine residues in proteins and nitrotyrosine formation has been used as a marker for the detection of the endogenous formation of peroxynitrite [45]. Using nitrotyrosine as a marker for the presence of ONOO- has been challenged by the demonstration that other reactions can also induce tyrosine nitration; e.g., the reaction of nitrite with hypochlorous acid and the reaction of myeloperoxidase with hydrogen peroxide can lead to the formation of nitrotyrosine [46]. Thus, increased nitrotyrosine staining is considered, as an indicator of "increased nitrosative stress" rather than a specific marker of the generation of peroxynitrite [47]. We have found that nitrotyrosine is indeed present in lung sections taken after carrageenan injection and that green tea extract reduced the staining in these tissues.

ROS and peroxynitrite produce cellular injury and necrosis via several mechanisms including protein denaturation, and DNA damage ROS produce strand breaks in DNA that trigger energy-consuming DNA repair mechanisms and activate the nuclear enzyme PARS, resulting in the depletion of its substrate NAD in vitro and a reduction in the rate of glycolysis. As NAD functions as a cofactor in glycolysis and the tricarboxylic acid cycle, NAD depletion leads to a rapid fall in intracellular ATP. This process has been termed the 'PARS suicide hypothesis'. There is recent evidence that the activation of PARS may also play an important role in inflammation [48,49].

We demonstrate here that green tea extract treatment reduced the activation of PARS during carrageenan-induced pleurisy in the lung. In light of the role of PARS in inflammation, it is possible that PARS inhibition by green tea extract accounts for the anti-inflammatory response.

Besides attenuating ONOO- production and PARS activation, green tea extract also reduced the development of oedema, neutrophil accumulation and had an overall protective effect on the degree of lung injury as assessed by histological examination.

A possible mechanism by which green tea extract attenuates PMNs infiltration is by down-regulating adhesion molecules ICAM-1

The activation and expression of adhesion molecules allows for the adhesion, conformational change, and extravasation (emigration) of the neutrophil that may induce local injury and participate in the orchestration of systemic inflammation and all of its consequences. ICAM-1 in particular plays a role in inflammatory processes and in the T cell-mediated host defense system. Within the endothelium, ICAM-1 has an important role in migration of leukocytes to sites of inflammation, enabling the firm adhesion and diapedesis of leukocytes. In accordance with these findings, we observed that green tea extract reduce the upregulation the surface expression of ICAM-1 on endothelial cells and prevents the infiltration of neutrophils at inflamed sites.

Lung inflammation is associated with enhanced expression of proinflammatory cytokines which serve as intercellular signals that recruit cells and modulate cell function. Cytokines produced predominantly by activated macrophages and lymphocytes mediate many inflammatory processes [49]. These proinflammatory cytokines include interferon-γ (IFN-γ), interleukin-1 (IL-1), tumour necrosis factor-α (TNFα) and chemokines (e.g. interleukin-8 [IL-8], macrophage chemotactic and activating factor [MCAF]). Though all of these cytokines play important roles in the evolving inflammatory response, TNFα appears to be a critical mediator of the inflammatory cascade. Numerous studies show that TNFα rises rapidly following acute trauma/inflammation/infection and that blocking TNFα activity reduces injury [50-52]. Recently it has been shown that, EGCG inhibits okadaic acid-induced TNFα production and gene expression in BALB/3T3 cells [53]. In agreement with these observations we have found in this study that green tea pre-treatment reduce exudates level of TNF-alpha. Moreover cytokines activate, several signaling pathways, leading to the downstream activation of NF-κB transcription factors. In this study we have placed our attention on STAT-1. Signal transducers and activators of transcription (STAT) factors are a family of cytoplasmic transcription factors that mediate intracellular signaling initiated at cytokine cell surface receptors and transmitted to the nucleus. Recently, it has been reported that the polyphenolic agent epigallocatechin-3-gallate (EGCG), a major constituent of green tea, is a potent inhibitor of STAT-1 phosphorylation and activation [26]. The JAK/STAT pathway has been shown to be essential for human and murine iNOS expression [24] and in ICAM-1 induction [25]. We demonstrate here that green tea extract pretreatment reduced STAT-1 activation in carrageenan-treated mice. The fall in STAT-1 activity may further account for the repression of ICAM-1 and iNOS lung expression with decrease in nitrite/nitrate concentration in the pleural exudate. Thus, the down-regulation of STAT-1 action provides a molecular basis for the anti-inflammatory effect of GTE.

Data generated from the present study indicate that green tea extract cause a substantial reduction of carraggenan induced-pleurisy in the mice suggesting toxicity from oxygen metabolites, released by stimulated neutrophils, macrophages, and other cells, as one of the significant mechanisms of lung injury. These findings support the potential use of green tea extract as therapeutic agents in the therapy of conditions associated with acute inflammation.
